# Effect of compressive stress on cavitation erosion-corrosion behavior of nickel-aluminum bronze alloy

**DOI:** 10.1016/j.ultsonch.2022.106143

**Published:** 2022-08-28

**Authors:** Zhenbo Qin, Xuehan Li, Da-hai Xia, Yiwen Zhang, Chao Feng, Zhong Wu, Wenbin Hu

**Affiliations:** a Tianjin Key Laboratory of Composite and Functional Materials, School of Material Science and Engineering, Tianjin University, Tianjin 300072, China; b Key Laboratory of Advanced Ceramics and Machining Technology (Ministry of Education), Tianjin University, Tianjin 300072, China; c State Grid Hunan Electric Power Company Limited Research Institute, Changsha 410007, China

**Keywords:** Compressive stress, Nickel-aluminum bronze, Cavitation erosion-corrosion, Synergistic effect

## Abstract

The effect of compressive stress on cavitation erosion-corrosion behavior of nickel-aluminum bronze alloy was investigated, and the results showed that the alloy exhibited selective phase corrosion of eutectoid “α + κ_iii_” and its destruction was aggravated with more cavitation mass loss up to 1.74 times of the specimen without stress. It was mainly owing to the enhanced corrosion-induced erosion caused by compressive stress, which led to lattice distortion of the alloy and the resulting accelerated selective phase corrosion with increasing surface roughness, and then intensified the synergistic effect of electrochemical corrosion and mechanical erosion.

## Highlights

• Loading compressive stress deteriorated the cavitation erosion-corrosion resistance.
• The synergetic mass loss (W*s*) remarkably increased with loading compressive stress.
• The applied of compressive stress has accelerated the corrosion of eutectoid “α + κ”.
• W*_CIE_* became the dominant factor of cavitation erosion-corrosion with loading stress.

## Graphical abstract

**Figure:**
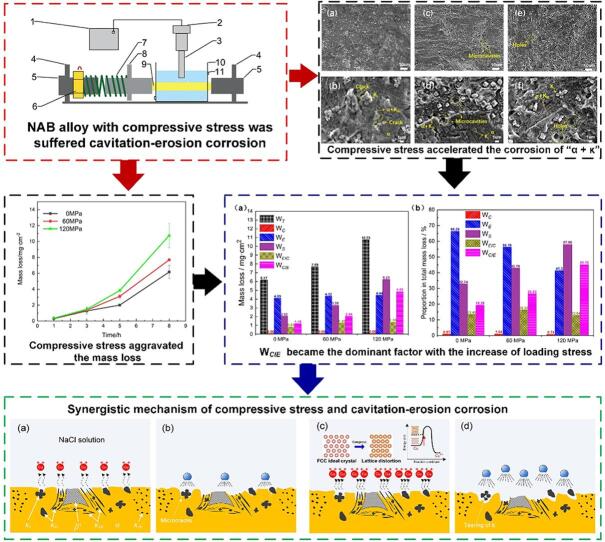

## 1 Introduction

Nickel-aluminum bronze (NAB) alloy is a Cu-based alloy that is widely used for marine propellers owing to its excellent comprehensive performance including of corrosion resistance, mechanical property and biological fouling resistance [1], [2], [3]. This alloy usually suffers from cavitation erosion-corrosion, which is a common phenomenon during material degradation by micro-jets or shock waves caused by the formation and collapse of bubbles attributing to the local pressure fluctuation in liquid [4], [5]. The cavitation erosion-corrosion behavior of NAB alloy has been investigated for decades, and it was found that α phase at the α/κ interface was cavitation corroded preferentially in neutral corrosion environment, leading to the formation of local cavities on the alloy surface [6], [7], [8]. However, these studies did not consider the effect of applied loads on cavitation behavior. In fact, the alloy used as blade is subjected to multiple forces when propellers are working, such as self-gravity, centrifugal force and shock stress induced by hydrodynamic fluid [9]. It has been proved that the equivalent stresses on the blade depended on the fluid hydraulic load and their values around propeller turbine runner could reach 14.75 MPa, 59.54 MPa and 75.132 MPa at the flow velocity of 1 m/s, 1.5 m/s and 2.25 m/s, respectively [9], [10], [11]. As is known to all, the applied stress could affect the corrosion resistance of the alloy [12], [13], which played an important role during the cavitation erosion-corrosion process [4], [5]. Therefore, the presence of applied stress would inevitably affect the cavitation erosion-corrosion behavior of the NBA alloy and its influence must be considered for the practical and objective valuation of cavitation erosion-corrosion performance.

So far, there is no relevant research related with the effect of elastic stress (less than the yield strength of materials) on cavitation erosion-corrosion process, and previous studies were mainly focused on its influence on static corrosion behavior of metallic materials. Some of them found that both tensile and compressive stresses deteriorated the corrosion product film, caused the negative shift of corrosion potential and the resulting accelerated corrosion of steel [14], [15], which was attributed to the intergranular stress corrosion cracking phenomenon induced by stress [16]. Besides, others confirmed that only tensile stress could aggravate corrosion by exposing porous corrosion product film beneficial for the diffusion of Cl^−^ to the metal matrix [17], while compressive stress was proved to retard corrosion by inhibiting the crack propagation and [18]. It could be concluded that the acceleration effect of tensile stress on corrosion process has been recognized unanimously, and the focus of argument was the function of compressive stress on corrosion behavior. Therefore, it is meaningful to evaluate the effect of compressive stress on cavitation erosion-corrosion behavior. However, coupling effect of compressive stress and cavitation erosion-corrosion on the degradation mechanism of the NAB alloy has not been reported.

In this work, the NAB alloy was subjected to different compressive stresses, and their corresponding cavitation erosion-corrosion behavior was studied by mass loss measurement, surface microstructure observation and electrochemical analysis. The degradation mechanism of the NAB alloy used as propellers was proposed by means of the synergistic effect of compressive stress and cavitation erosion-corrosion.

## 2 Experimental

### 2.1 Material

**Fig. 1:**
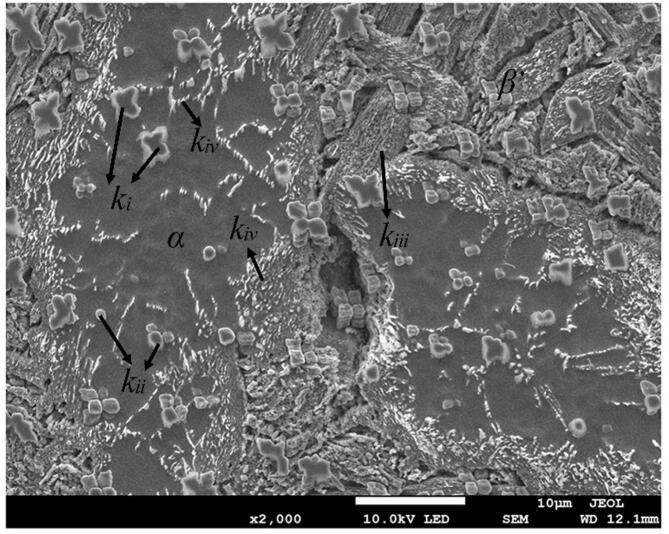
Microstructures of an as-cast NAB alloy.

An as-cast NAB alloy was selected as the experimental material. The chemical composition determined by X-ray fluorescence spectrum was 78.8 wt% Cu, 11.58 wt% Al, 3.98 wt% Ni, 5.12 wt% Fe, 0.43 wt% Zn and 0.09 wt% Mn. Then it was cut into specimens with a dimension of 40*10*5 mm for the following study. The alloy was consisted of a copper-rich α matrix phase, a martensitic β′ phase and intermetallic κ*_i_*, κ*_ii_*, κ*_iii_* and κ*_iv_* phases [19], [20], [21], as depicted in Fig. 1.

### 2.2 Loading of compressive stress

**Fig. 2:**
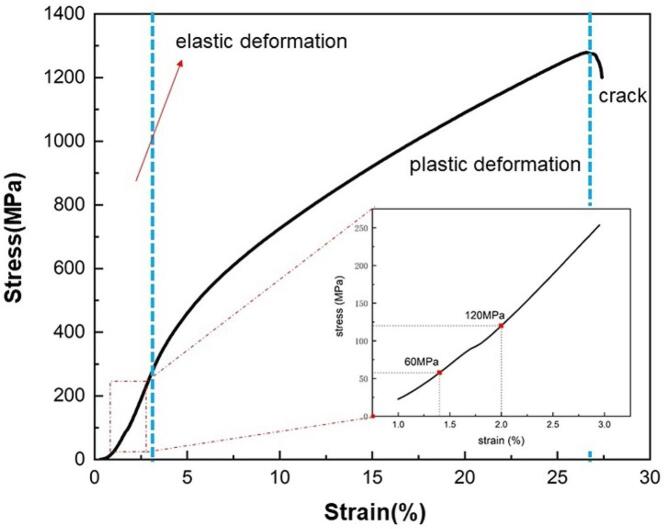
Stress–strain curve of the NAB alloy under compressive stress.

**Fig. 3:**
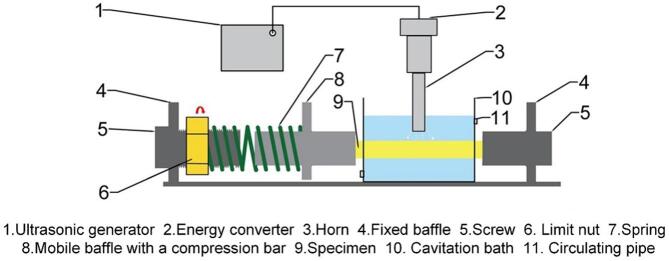
Schematic diagram of compressive stress loading device used in cavitation erosion-corrosion test.

According to the compressive stress–strain curve shown in Fig. 2, stresses of 60 MPa and 120 MPa in the elastic deformation zone were selected to apply on the NAB alloy. A compressive stress loading device was designed in cavitation erosion-corrosion test, as shown in Fig. 3. The compressive stress was applied by adjusting the spacing between three steel baffles made of 304 stainless steels. Among them, the baffles on both sides were fixed, while the baffle in the middle could be moved left or right. By rotating the limited nut, the spring was compressed, pushing the middle baffle close to the right baffle. As a result, the specimen placed between the two baffles was subjected to compressive stress and generated compressive deformation. Because the spring was rigidly connected with the baffle, the force applied to the specimen could be calculated by the deformation of the spring. Considering that the elastic modulus of 304 stainless steel (200 GPa) is much greater than that of NAB alloy (127 GPa) [22], [23], [24], the deformation of baffle plate was ignored. The cross-sectional area of the specimen was 50 mm^2^and the stiffness coefficient of the spring was 329.67 N/mm. Therefore, the spring was compressed by 9.1 mm and 18.2 mm to achieve the compressive stresses of 60 MPa and 120 MPa on the specimen, respectively.

### 2.3 Cavitation erosion-corrosion tests

Cavitation erosion-corrosion tests were performed on specimens loading with compressive stress with an ultrasonic vibration apparatus following ASTM G32 standard [26]. Prior the test, specimens were pretreated by grinding, mechanical polishing to mirror-grade, and then cleaned with anhydrous ethanol in an ultrasonic cleaning machine. During the test, the specimens were located coaxially with the horn and held 0.5 mm from the ultrasonic horn, which operated at a frequency of 20 kHz and an amplitude of 45 μm. 3.5 wt% NaCl solution and distilled water were selected as the test medium to explore the contribution of electrochemical corrosion and mechanical impact. The temperature was maintained at about 25 ± 1 ℃ using the cooling water. After the test, the specimens were cleaned, dried, and weighed by an analytical balance with accuracy of 0.01 mg to measure the mass loss. Tests were repeated at least three times to ensure the accuracy. Both the surface and sectional structure after cavitation erosion-corrosion test was observed by scanning electron microscope (SEM).

### 2.4 Electrochemical measurements

Electrochemical measurements were conducted through an electrochemical system (Ivium-Vertex. One) in neutral 3.5 wt% NaCl solution at 25℃, with a saturated calomel electrode and a platinum plate served as the reference and counter electrode, respectively. Specimens were sealed by waterproof tape, leaving an area of 1 cm^2^ exposed and then conducted on electrochemically test in 3.5 wt% NaCl solution under quiescent or cavitation condition.

Firstly, open circuit potential (OCP) was continuously recorded under the quiescent condition until the potential fluctuates less than 5 mV within 5 min [25]. Then electrochemical impedance spectroscopy (EIS) measurements were carried out at OCP from 100 kHz to 10 mHz with a potential perturbation of 5 mV. After that, linear polarization resistance was measured in the range of ± 5 mV around OCP at the sweep speed of 0.33 mV/s. Subsequently, potentiodynamic polarization curves were measured at a rate of 0.5 mV/s and corrosion current density was obtained by Tafel fitting of the cathode polarization curve. The selected linear region was from − 100 mV to − 150 mV versus corrosion potential [26]. The corrosion current density was converted into corrosion mass loss by Faraday's law [27], [28]:

(1) $$\text{Mass loss rate}=\frac{I_{\text{corr}}\times M\times S}{n\times F}$$

where *M* and *n* represent atomic mass and number of free electrons, respectively. As the content of copper in NAB alloy is around 80 wt%, its corrosion process is mainly the oxidation reaction of copper, and thus the values of *M* and *n* are 63.5 and 2, respectively. *S* refers to the exposed area with the value of 1 cm^2^. *F* is Faraday’s constant (96,485C/mol).

## 3 Results

### 3.1 Cavitation erosion-corrosion performance with compressive stress

**Fig. 4:**
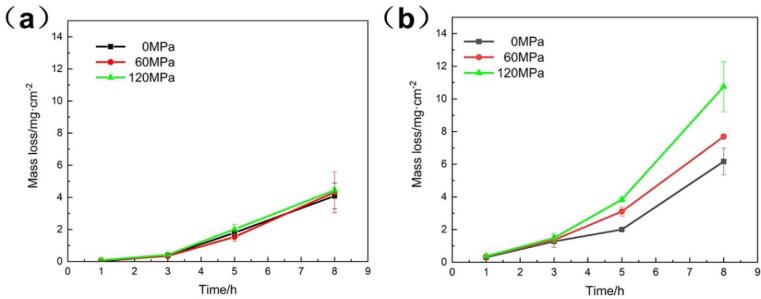
Cavitation mass loss of the NAB alloy with different compressive stresses in (a) distilled water and (b) 3.5 wt% NaCl solution.

The cavitation erosion-corrosion damage can be directly evaluated by total mass loss of the metals [29]. Fig. 4 shows the mass loss as a function of cavitation erosion-corrosion time for the specimens in distilled water and 3.5 wt% NaCl solution and the detailed results could be seen in Table 1. It could be found in Fig. 4a that there was no obvious difference in the mass loss in the distilled water with different compressive stresses, indicating that the compressive stress had little effect against the mechanical attack during cavitation process. In the NaCl solution (Fig. 4b), the mass loss was more than that of distilled water, attributed from the accelerated corrosion. What's more, it is obvious that the cavitation mass loss increased with compressive stress in NaCl solution with the value of 10.75 mg/cm^2^ under 120 MPa compressive stress, which was about 1.74 times as much as that of the specimen without loading stress (6.17 mg/cm^2^).

**Table 1:** Cavitation mass loss of the NAB alloy with different compressive stresses in distilled water and 3.5 wt% NaCl solution.

Compressive stress	Distilled water			3.5 wt% NaCl		
	0 MPa	60 MPa	120 MPa	0 MPa	60 MPa	120 MPa
1 h	0.02 (±0.01)	0.07 (±0.01)	0.08 (±0.04)	0.30 (±0.04)	0.36 (±0.16)	0.36 (±0.02)
3 h	0.37 (±0.14)	0.35 (±0.03)	0.42 (±0.04)	1.27 (±0.37)	1.36 (±0.06)	1.48 (±0.16)
5 h	1.79 (±0.15)	1.53 (±0.26)	2.01 (±0.3)	2.00 (±0.06)	3.11 (±0.28)	3.83 (±0.14)
8 h	4.09 (±0.80)	4.31 (±1.20)	4.44 (±0.20)	6.17 (±0.82)	7.69 (±0.16)	10.75 (±1.53)

### 3.2 Cavitation erosion-corrosion morphology with compressive stress

**Fig. 5:**
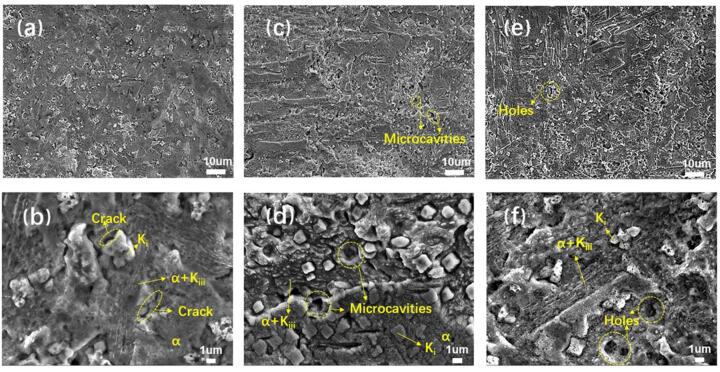
Surface micrographs of the NAB alloy with (a, b) 0 MPa, (c, d) 60 MPa and (e, f) 120 MPa compressive stress after cavitation corroded for 3 h in 3.5 wt% NaCl solution.

**Fig. 6:**
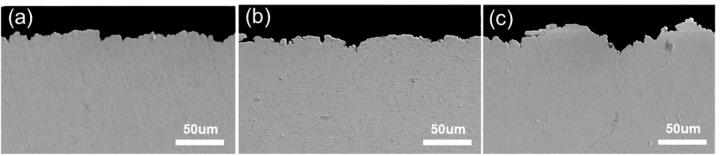
Cross-sectional micrographs of the NAB alloy with (a) 0 MPa, (b) 60 MPa and (c) 120 MPa compressive stress after cavitation corroded for 8 h in 3.5 wt% NaCl solution.

According to Fig. 4b, the mass loss of the specimens with different compressive stresses began to show a slight difference when 3-hour cavitation in the NaCl solution. Then these specimens were selected to observe their cavitation corroded surface morphology by SEM, as shown in Fig. 5. The surface of NAB alloy without loading stress was slightly damaged by cavitation erosion-corrosion (Fig. 5a and b), with eutectoid “α + κ*_iii_*” phase prone to be corroded preferentially. While, the hard phase κ, as the cathode phase in neutral corrosive medium [30], suffered less cavitation damage to be exposed on the alloy surface. And cracks were found at the α/κ phase boundary, attributed to the repeated impact of cavitation pulse stress. This result has also been confirmed by our previous research [31]. When the specimen was subjected to the compressive stress of 60 MPa, microcavities appear on the surface, as identified in Fig. 5c. At a higher magnification (Fig. 5d), the formation of microcavities was induced by the dissolution of α phase around κ ones, which destroyed the structural integrity and led to the shedding of κ phase. Meanwhile, “α + κ*_iii_*” eutectoid structure was damaged more seriously by cavitation, indicating that the compressive stress accelerated the dissolution of α phase at α/κ interface and aggravated the destruction of the eutectoid structure. This phenomenon becomes more obvious when the stress reached 120 MPa, as shown in Fig. 5e and f. Cavities caused by κ phase shedding became larger, and the destruction of eutectoid tissue generated deep holes. With the increase of cavitation time, its damage conducted on the specimen became more serious, led to the difficulty of distinguishing the alloy microstructure from the surface topography. Thus, the cross-sectional morphologies after 8-hour cavitation in the NaCl solution were observed, as shown in Fig. 6. It is clearly that the compressive stress deepened the cavities and increased the surface roughness.

### 3.3 Electrochemical properties coupled of cavitation erosion-corrosion and compressive stress

**Fig. 7:**
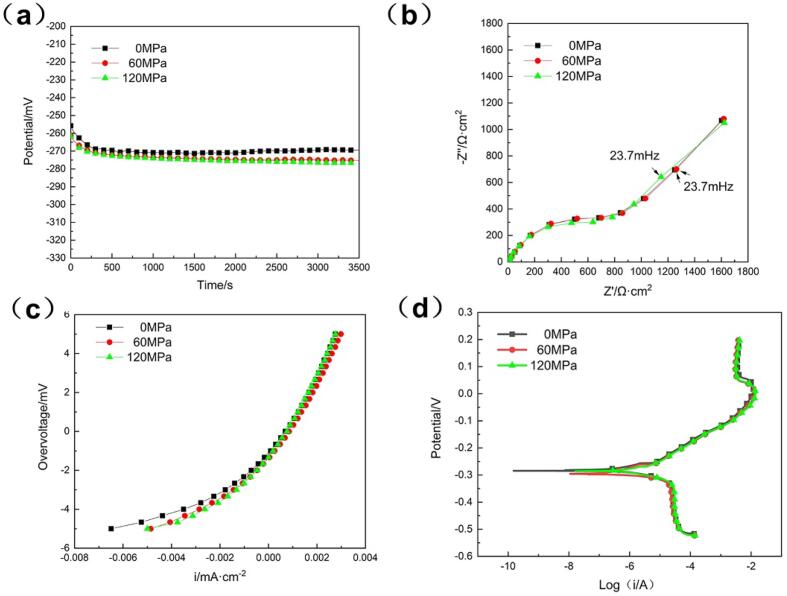
(a) Open circuit potential, (b) Nyquist plots, (c) linear polarization plots and (d) potentiodynamic polarization curves of the NAB alloy with different compressive stresses in quiescent condition.

Fig. 7 shows the electrochemical properties of the NAB alloy with different compressive stresses in quiescent condition. OCP, Nyquist plots, linear polarization plots and polarization curves almost overlap with each other for all specimens, indicating that there was little effect of elastic compressive stress on the static corrosion behavior, which is also consistent with our previous findings [32]. Besides, all the Nyquist plots exhibit a single semicircle and a diffusive Warburg straight line, which signifies that the reaction was controlled by the diffusion of oxygen [33].

**Fig. 8:**
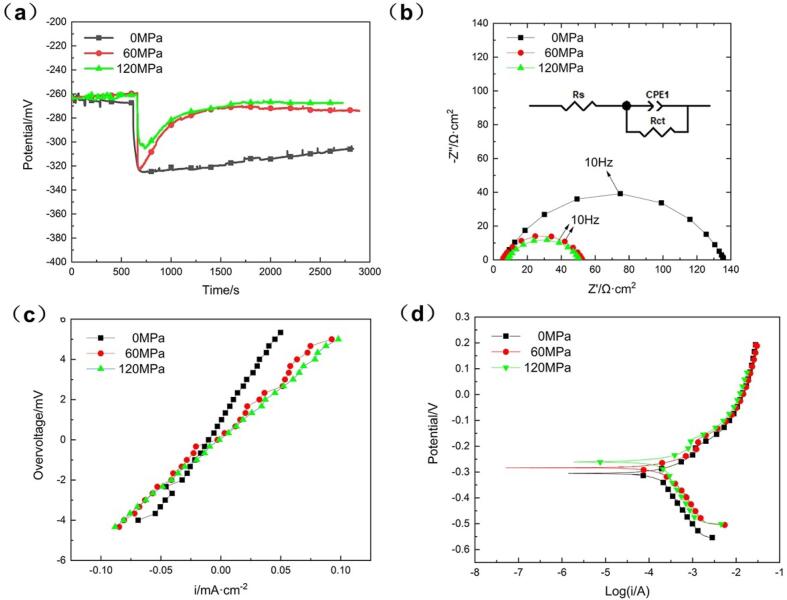
(a) Open circuit potential, (b) Nyquist plots, (c) linear polarization plots and (d) potentiodynamic polarization curves of the NAB alloy with different compressive stress in cavitation erosion-corrosion condition.

While, for the cavitation erosion-corrosion condition, significant difference in the electrochemical properties could be found in Fig. 8. There is 10–60 mV negative shift of OCP induced by cavitation erosion-corrosion as shown in Fig. 8a, attributed to the removal of protective product film on the alloy surface to expose the fresh surface [28], [34]. It should be noted that the OCP of the alloy subjected to compressive stress moved in the positive direction subsequently, and reached the stable value with about 35 mV higher than that of the one without loading stress, owing to the effect of compressive stress on its corrosion behavior [18] and would be discussed later in Section 4.2. All Nyquist plots during cavitation erosion-corrosion exhibit a single semicircle without Warburg straight line (Fig. 8b), owing to the explosion of bubbles to bring large amount of dissolved oxygen and the resulting reaction free from the limitation of oxygen diffusion [28]. Moreover, the semicircle diameter for the specimens with compressive stress was reduced markedly, indicating the deterioration of corrosion resistance. An equivalent circuit model was utilized to simulate the corrosion process (shown in the inset image of Fig. 8b), where R*_s_* as the solution resistance, R*_ct_* as the charge transfer resistance at the alloy/electrolyte interface, CPE as the non-ideal capacitance of charge transfer. Among them, CPE is defined by two parameters of CPE-T and CPE-P, and the former indicates the value of capacitance of the CPE element while the later accounts for the change of the compressed semicircle from an ideal one [35]. The fitted electrochemical parameters are listed in Table 2. Applied compressive stress significantly reduced the values of R*_ct_*, indicating that the electrons were easy to transfer with relatively lower oxidation reaction barrier [36], [37]. This could be attributed to the crystal lattice distortion induced by compressive stress, causing the metal atoms to deviate from the equilibrium position [38], [39]. In addition, the redox capacitance, CPE-T, increases gradually with the compressive stress, presumably due to the enlargement of the area involved in the electrochemical reaction under the compressive stress [40].

**Table 2:** Equivalent circuit parameter values determined by impedance spectra fitting analysis of the NAB alloy with different compressive stresses in cavitation erosion-corrosion condition.

Specimens	R*_s_* (Ω·cm^2^)	CPE-T (μF·cm^2^·s*^n^*^−1^)	CPE-P	R*_ct_* (Ω·cm^2^)
0 MPa	5.893	403.10	0.691	130.20
60 MPa	5.271	467.75	0.695	47.28
120 MPa	7.873	786.42	0.645	43.12

Fig. 8c shows the linear polarization curves, where the slope of curve (R*_p_*) decreased with the increasing compressive stress, indicating a gradual diminishing of corrosion resistance. Fig. 8d shows the Tafel curves under different stresses with a similar shape, implying the corrosion reaction mechanism of NAB did not change with the compressive stress. And the specimen with 120 MPa exhibits the highest value of E*_corr_*, consistent with the change tendency of OCP described aforementioned. By Tafel extrapolation, the calculated electrochemical corrosion parameters for both quiescent and cavitation erosion-corrosion condition are listed in Table 3. And the corrosion current density (I*_corr_*) increased with the compressive stress during cavitation erosion-corrosion. In particular, I*_corr_* for the specimen with 120 MPa stress (1.54 × 10^−4^ A/cm^2^) was about 1.6 times as much as that of the one without loading stress (0.94 × 10^−4^ A/cm^2^), while the relatively less increase of I*_corr_* in quiescent condition.

**Table 3:** Electrochemical parameters of the NAB alloy with different compressive stresses in quiescent and cavitation erosion-corrosion condition.

Compressive stress	Quiescent		Cavitation erosion-corrosion	
	E*_corr_* (V)	I*_corr_* (10^−6^A·cm^−2^)	E*_corr_* (V)	I*_corr_* (10^−6^A·cm^−2^)
0 MPa	−0.282	6.62	−0.305	94.4
60 MPa	−0.296	8.05	−0.284	139.8
120 MPa	−0.296	8.93	−0.261	154.0

## 4 Discussion

### 4.1 Synergistic effect of cavitation erosion-corrosion under compressive stress

The damage induced by Cavitation erosion-corrosion in marine environment is the synergistic result of electrochemical corrosion and mechanical impact, and the main factors include corrosion, mechanical erosion and their synergy [41]. It has been reported that quantifying the role of each factor during cavitation erosion-corrosion is an effective method to analyze its mechanism [42]. According to the criteria for synergistic effects during cavitation erosion-corrosion tests, ASTM G119-93 [43], the total mass loss (*W_T_*) of cavitation can be divided into the following sections [1], [25]:

(2) W*_T_* = W*_C_* + W*_E_* + W*_S_*

where W*_C_* and W*_E_* represent the mass loss caused by corrosion and mechanical erosion, respectively. W*_S_* is the mass loss induced by their synergy, and it can be divided into W*_CIE_* and W*_EIC_*. W*_CIE_* is the mass loss of corrosion-induced erosion, related with the fact that the corrosion reaction destroyed the surface structural integrity of the alloy and reduced its mechanical strength, easy to failure under the repeated impact of cavitation stress. W*_EIC_* is the mass loss of erosion-induced corrosion, which can be interpreted in two ways: 1) increase of ions diffusion by the detachment of protective film; 2) enhancement of oxygen supply by severe stirring. Thus, the total mass loss can be defined as:

(3) W*_T_* = W*_C_* + W*_E_* + W*_CIE_* + W*_EIC_*

The values of W*_T_* and W*_E_* were measured during cavitation erosion-corrosion in the NaCl solution and distilled water, respectively. W*_C_* was calculated from I*_corr_* in quiescent condition according to Eq. (1). And W*_EIC_* was obtained by the following equation:

(4) W*_EIC_* = W*_C′_*-W*_C_*

where W*_C′_* was the corrosion mass loss during cavitation erosion-corrosion, and it was also calculated from I*_corr_* in cavitation condition according to Eq. (1). The values of I*_corr_* in both quiescent and cavitation conditions are listed in Table 3.

**Fig. 9:**
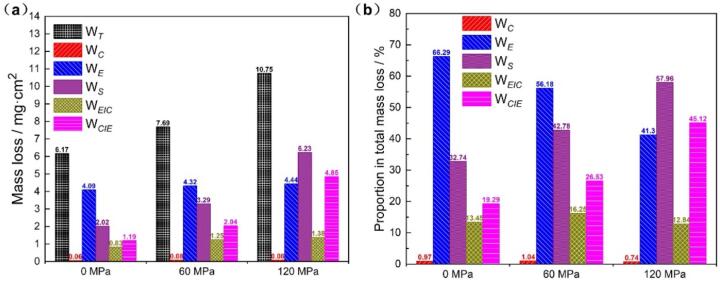
Mass loss (a) and the corresponding proportion (b) of corrosion (W*_C_*), erosion (W*_E_*), corrosion-induced erosion (W*_CIE_*), erosion-induced corrosion (W*_EIC_*) and total cavitation erosion-corrosion (W*_T_*) for the NAB alloy with different compressive stresses after 8-hour cavitation erosion-corrosion.

Values of W*_T_*, W*_C_*, W*_E_*, W*_EIC_* and W*_CIE_* and their proportions after 8-hour cavitation are shown in Fig. 9. It was obvious that, for all specimens, W*_E_* accounts for nearly half of the total mass loss (W*_T_*), indicating that the mechanical damage played a leading role in cavitation erosion-corrosion [1]. Moreover, the values of W*_C_* and W*_E_* almost remain constant, despite the alloy suffered compressive stress. However, the synergetic mass loss (W*_s_*) increased with the compressive stress. The value of W*_s_* for the compressed specimen with 120 MPa was 6.23 mg/cm^2^, which was nearly 3.1 times as much as that of specimen without loading stress (2.02 mg/cm^2^). That is to say, the applied compressive stress has not changed the corrosion behavior or the erosion resistance of the NAB alloy, but remarkably affected its mechano-chemical synergy in the process of cavitation erosion-corrosion. In particularly, the components of W*_S_*, both W*_EIC_* and W*_CIE_* increased with the applied stress. W*_EIC_* increased from 0.83 mg/cm^2^ to 1.25 mg/cm^2^ when loading 60 MPa, and then reached 1.38 mg/cm^2^ at 120 MPa, owing to the enhanced energy of the alloy atoms by applied compressive stress [36], [44]. The surface high-energy metal atoms were more prone to corrosion with the exfoliation of surface film and the increment of oxygen supply caused by ultrasonic cavitation oscillation. W*_CIE_* increased by three times from 1.19 mg/cm^2^ to 4.85 mg/cm^2^ when the applied stress up to 120 MPa. Accordingly, the proportion of W*_CIE_* account for W*_T_* also rose significantly from 19.4% to 45.2%, which has exceeded the value of W*_E_* and became the dominant factor during cavitation erosion-corrosion. The corrosion process was intensified by the applied compressive stress, to create more roughen alloy surface [45], led to more generation of cavitation cavities and the resulting enhanced mass loss [46].

Based on the above analysis, the applied compressive stress on the NAB alloy accelerated the mechano-chemical synergy during cavitation erosion-corrosion, which was mainly attributed to the strengthening of corrosion-induced mechanical damage. To be more specific, the presence of compressive stress intensified the corrosion reaction of the NAB alloy during cavitation, and consequently aggravated the destruction of cavitation erosion-corrosion. The effect of compressive stress on corrosion process during cavitation erosion-corrosion was further discussed by electrochemical analysis in Section 4.2.

### 4.2 Effect of compressive stress on corrosion process during cavitation erosion-corrosion

**Fig. 10:**
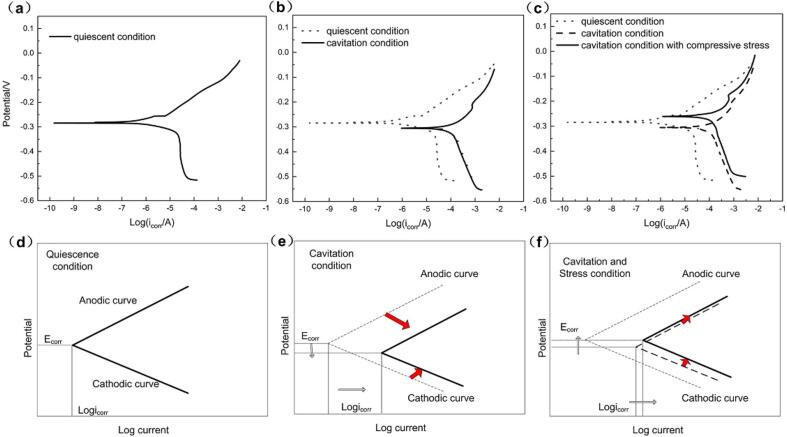
Experimental results of polarization curves in (a) quiescent condition, (b) cavitation erosion-corrosion condition, (c) cavitation erosion-corrosion condition with compressive stress and a simplified model of polarization curves in (d) quiescent condition, (e) cavitation erosion-corrosion condition and (f) cavitation erosion-corrosion condition with compressive stress.

A simplified model (shown in the Fig. 10-d, e and f) according to polarization curves (shown in the Fig. 10-a, b and c) was proposed to evaluate the corrosion process of the NAB alloy with or without loading stress in quiescence and cavitation condition, as shown in Fig. 10. The anodic reaction of the alloy is mainly the dissolution of Cu as follows [47]:

(5) Cu + 2Cl^−^ → CuCl_2_^−^ + e^−^

The cathodic reaction comes from the oxygen reduction [1], [32]:

(6) O_2_ + 2H_2_O + 4e^−^ → 4OH^−^

Compared with the electrochemical reaction of NAB alloy in quiescent condition, mechanical shock during cavitation erosion-corrosion removed the protective film covering on the alloy surface, resulting in the exposure of fresh metal to the corrosive medium and enhancing the anodic reaction of Cu dissolution. Besides, the cathodic reaction of oxygen reduction was also accelerated by increased mass transfer of dissolved oxygen from the solution agitation caused by cavitation erosion-corrosion. Therefore, both the anodic and cathodic branches of the polarization curve measured during cavitation erosion-corrosion shifted in the direction of high current, as shown in Fig. 10a-b and d-e. According to Fig. 7 and Fig. 8, both OCP and E*_corr_* of the alloy without loading stress moved negatively in cavitation erosion-corrosion condition, indicating that the shift magnitude of anodic branch was larger than that of cathodic branch, as marked by the arrow in Fig. 10e. And thus it could be inferred that, the detachment of protective film played a more important role than that of the enhancement oxygen supply for the electrochemical reaction of the NAB alloy during cavitation erosion-corrosion [28].

When the NAB alloy conducted on cavitation erosion-corrosion was subjected to compressive stress, the polarization curves shifted further, which can be clearly observed in Fig. 8d. The cathodic branch shifted towards the direction of current increase, owing to the roughen surface and the resulting surface pressure fluctuation induced by the loading compressive stress [48], [49], [50], which promoted the generation of cavitation bubbles and brought more oxygen to accelerate the oxygen reduction reaction on the cathode [46]. While, it is difficult to distinguish the change of corrosion current from the anodic branches. Therefore, the current values under the same potential for specimens with different compressive stresses were extracted from the curves and listed in Table 4. It could be found that the anodic current shows an increasing trend with compressive stress. The corresponding simplified model was expressed in Fig. 10f that the anodic branch appears to move to the upper left, indicating the accelerated anodic reaction. This can be attributed to the lattice distortion of the alloy [38], [39] caused by loading compressive stress that raised the energy of metal atoms [36], which promotes the dissolution of copper. Since the surface roughness became larger with cavitation time, more bubbles generated and the cathodic reaction was accelerated further. It could be concluded that the compressive stress had a greater impact on the cathodic reaction than that of the anodic one during cavitation erosion-corrosion process, exhibiting the shift of E*_corr_* in the positive direction. In conclusion, applied compressive stress promoted the corrosion of the NAB alloy and the resulting enhanced corrosion-induced erosion (*W_CIE_*) effect, which became the dominant factor during cavitation erosion-corrosion with the proportion account for W_T_ of 45.2% at the compressive stress of 120 MPa.

**Table 4:** Polarization current densities (I) of different compressive stresses under the different overpotential.

Compressive stress	E*_corr_* + 50 mV		E*_corr_* + 100 mV		E*_corr_* + 200 mV	
	Log I	I (10^−6^A/cm^2^)	Log I	I (10^−6^A/cm^2^)	Log I	I (10^−6^A/cm^2^)
0 MPa	−3.38	418	−3.09	802	−2.35	4491
60 MPa	−3.27	536	−3.05	887	−2.30	5024
120 MPa	−3.23	588	−2.87	1350	−3.27	5858

### 4.3 Cavitation erosion-corrosion behavior of NAB alloy with compressive stress

**Fig. 11:**
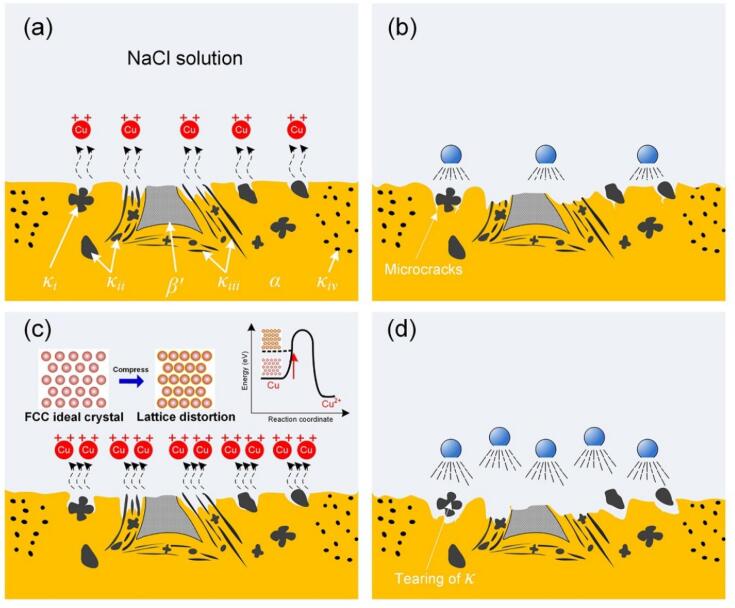
Schematic of the cavitation erosion-corrosion behavior of the NAB alloy in NaCl solution (a, b) without and (c, d) with compressive stress.

The cavitation erosion-corrosion behavior of the NAB alloy in the NaCl solution was proposed, as schematically indicated in Fig. 11. When the alloy suffered cavitation erosion-corrosion (Fig. 11a and b), it was not only electrochemical corroded by the corrosive medium, but also mechanical damaged by the cavitation microjets and shock waves. The disparity of relative nobility for each phase in corrosive medium resulted in the selective phase corrosion, i.e. α was the anodic phase compared to β′ and κ phases. And α adjacent to κ precipitates were prone to being corroded preferentially. In particular, due to the lamellar spacing distribution of eutectoid “α + κ*_iii_*”, the dissolution of α phase was more rapid along the depth direction, as shown in Fig. 11a. The corrosion reaction caused an incomplete surface structure and reduced its mechanical strength. Under the repeated impact of cavitation erosion-corrosion, microcracks appeared at the boundary of α/κ phase, and then κ phase fell off subsequently, resulting in mass loss of the NAB alloy. Due to the synergistic effect of corrosion and mechanical impact, the damage of eutectoid “α + κ*_iii_*” was more obvious with deeper cavities, as depicted in Fig. 11b.

For the NAB alloy loading with compressive stress (Fig. 11c and d), lattice distortion increased the atomic energy, and the atoms on the alloy surface were exposed to the corrosive medium with accelerated oxidation reaction when subjected to cavitation erosion-corrosion. Therefore, the corrosion attack of α phase adjacent to κ one was aggravated, and the surface structural imperfection of the alloy as well as its reduction in the mechanical strength were more obvious compared with the condition without loading stress, as can be seen in Fig. 11c. Subsequently, κ phase spalled against the mechanical impact of cavitation with larger and deeper cavities in eutectoid “α + κ*_iii_*”, resulting in higher surface roughness (shown in Fig. 5 and Fig. 6), which led to the inhomogeneous distribution of elastic energy on the alloy surface with larger elastic energy stored at the valleys compared with that at the plane [51]. Therefore, the energy of atoms in the cavities was excited higher with raised corrosion rate induced by loading compressive stress, further leading to more serious local corrosion and higher surface roughness, as illustrated in Fig. 11d. This was conducive to the generation of cavitation bubbles, and the blasting of extra bubbles increased the damage to the alloy with more mass loss (*W_T_*). In summary, the corrosion process of the NAB alloy was promoted by applied compressive stress and then enhanced the corrosion-induced erosion (*W_CIE_*) effect, which acted as the key factor during cavitation erosion-corrosion coupled with loading compressive stress.

## 5 Conclusions

(1) Compressive stress deteriorated the cavitation erosion-corrosion resistance of the NAB alloy. The mass loss increased with the compressive stress. After cavitation erosion-corrosion for 8 h, the mass loss for the specimen with 120 MPa compressive stress was 10.75 mg/cm^2^, which was about 1.74 times as much as that of the specimen without loading stress.
(2) The synergetic mass loss (W*_s_*) increased significantly with the loading compressive stress, and the value at 120 MPa was 6.23 mg/cm^2^, which was almost 3.1 times as much as that of the specimen without loading stress (2.02 mg/cm^2^). Its components, both W*_EIC_* and W*_CIE_* increased with the applied stress. It should be noted that the proportion of W*_CIE_* account for W*_T_* rose significantly from 19.4% to 45.2%, which has exceeded the value of W*_E_* and became the dominant factor during cavitation erosion-corrosion owing to the enhanced corrosion-induced erosion effect.
(3) Applied compressive stress has not changed the selective phase corrosion behavior of the NAB alloy during cavitation erosion-corrosion, where α phase adjacent to κ was preferentially subjected to cavitation erosion-corrosion, regardless of compressive stress. However, compressive stress has accelerated the corrosion process and led to the formation of large cavities in the eutectoid “α + κ_*iii*_”. It has been further confirmed by the electrochemical measurements, where the NAB alloy exhibited relatively smaller R*_ct_* and higher I*_corr_* during cavitation erosion-corrosion with loading compressive stress.
(4) The cavitation erosion-corrosion behavior of the NAB alloy with loading compressive stress was proposed. Compressive stress caused lattice distortion of the alloy and accelerated the selective corrosion of α phase. The intensified localized corrosion reduced the alloy strength, forming surface cavities and causing an increase in roughness, which facilitated the generation of cavitation bubbles with serious damage against the alloy. The corrosion induced erosion (W*_CIE_*) effect enhanced by loading compressive stress has become the dominant factor during cavitation erosion-corrosion of the NAB alloy.

## Declaration of Competing Interest

The authors declare that they have no known competing financial interests or personal relationships that could have appeared to influence the work reported in this paper.

## Data Availability

The raw/processed data required to reproduce these findings cannot be shared at this time due to technical limitations.

## References

[1] Luo Q., Zhang Q., Qin Z.B., Wu Z., Shen B., Liu L., Hu W.B. (2018). The synergistic effect of cavitation erosion and corrosion of nickel-aluminum copper surface layer on nickel-aluminum bronze alloy. J. Alloy. Compd..

[2] Zhang B.B., Wang J.Z., Yan F.Y. (2018). Load-dependent tribocorrosion behaviour of nickel-aluminium bronze in artificial seawater. Corros. Sci..

[3] Ahmad A., Li H.J., Pan Z.X., Cuiuri D., Van Duin S., Larkin N., Polden J., Lane N. (2014). Feasibility study of low force robotic friction stir process and its effect on cavitation erosion and electrochemical corrosion for Ni Al bronze alloys. Metall. Mater. Trans. B-Process Metall. Mater. Process. Sci..

[4] Tang C.H., Cheng F.T., Man H.C. (2004). Effect of laser surface melting on the corrosion and cavitation erosion behaviors of a manganese-nickel-aluminium bronze. Mater. Sci. Eng. A-Struct. Mater. Propert. Microstruct. Process..

[5] Wang L.Q., Qiu N., Hellmann D.H., Zhu X.W. (2016). An experimental study on cavitation erosion-corrosion performance of ansi 1020 and ansi 4135 steel. J. Mech. Sci. Technol..

[6] Song Q.N., Zheng Y.G., Jiang S.L., Ni D.R., Ma Z.Y. (2013). Comparison of corrosion and cavitation erosion behaviors between the as-cast and friction-stir-processed nickel aluminum bronze. Corrosion.

[7] Song Q.N., Zheng Y.G., Ni D.R., Ma Z.Y. (2014). Corrosion and cavitation erosion behaviors of friction stir processed Ni-Al bronze: Effect of processing parameters and position in the stirred zone. Corrosion.

[8] Al-Hashem A., Riad W. (2002). The role of microstructure of nickel–aluminium–bronze alloy on its cavitation corrosion behavior in natural seawater. Mater. Charact..

[9] Yu K.Y. (2020). Peikai;Hu, Jian., Numerical analysis of blade stress of marine propellers. J. Mar. Sci. Appl..

[10] Waqas M., Ahmad N. (2020). Computation of stress distribution in hydraulic horizontal propeller turbine runner based on fluid-structure interaction analysis. Arab. J. Sci. Eng..

[11] Md M.P.H., Mezbah Uddin Md, Jahan Morshed, Iftekharul Islam Md (2021). Structural analysis of composite propeller of ship using fem. AIP Conf. Proc..

[12] Yang H.Q., Zhang Q., Tu S.S., Wang Y., Li Y.M., Huang Y. (2016). Effects of inhomogeneous elastic stress on corrosion behaviour of Q235 steel in 3.5% NaCl solution using a novel multi-channel electrode technique. Corros. Sci..

[13] Yang H.Q., Zhang Q., Tu S.S., Wang Y., Li Y.M., Huang Y. (2016). A study on time-variant corrosion model for immersed steel plate elements considering the effect of mechanical stress. Ocean Eng..

[14] Feng X.G., Lu X.Y., Zuo Y., Zhuang N., Chen D. (2016). Electrochemical study the corrosion behaviour of carbon steel in mortars under compressive and tensile stresses. Corros. Sci..

[15] Wang X.H., Tang X.H., Wang L.W., Wang C., Zhou W.Q. (2014). Synergistic effect of stray current and stress on corrosion of api x65 steel. J. Nat. Gas Sci. Eng..

[16] Dollah M., Robinson M.J. (2011). Stress corrosion cracking of aluminium alloy 7075(w) under tensile and compressive loading. Corros. Eng., Sci. Technol..

[17] Zhang S., Pang X.L., Wang Y.B., Gao K.W. (2013). Corrosion behavior of steel with different microstructures under various elastic loading conditions. Corros. Sci..

[18] Zhang Y., Poursaee A. (2015). Passivation and corrosion behavior of carbon steel in simulated concrete pore solution under tensile and compressive stresses. J. Mater. Civ. Eng..

[19] Lv Y.T., Wang L.Q., Han Y.F., Xu X.Y., Lu W.J. (2015). Investigation of microstructure and mechanical properties of hot worked nial bronze alloy with different deformation degree. Mater. Sci. Eng. A-Struct. Mater. Propert. Microstruct. Process..

[20] Park K.S., Kim S. (2011). Corrosion and corrosion fatigue characteristics of cast nab coated with nab by hvof thermal spray. J. Electrochem. Soc..

[21] Muller S., Wolverton C., Wang L.W., Zunger A. (2001). Prediction of alloy precipitate shapes from first principles. Europhys. Lett..

[22] Okoro A.M., Lephuthing S.S., Rasiwela L., Olubambi P.A. (2021). Nondestructive measurement of the mechanical properties of graphene nanoplatelets reinforced nickel aluminium bronze composites. Heliyon.

[23] Ma G., Ling X. (2011). Study on hardness and elastic modulus of surface nanostructured 304 stainless steel using two mechanical methods. J. Pressure Vessel Technol. Trans. ASME.

[24] Song H.Q., Zhang L.Y., Shen H.X., Cao F.Y., Zhao X.Y., Gu X., Jin Z.S., Sun J.F. (2021). Morphology and fracture of oxide bifilm defects in nickel-aluminium bronze. J. Mater. Res. Technol..

[25] Basumatary J., Wood R.J.K. (2020). Different methods of measuring synergy between cavitation erosion and corrosion for nickel aluminium bronze in 3.5% nacl solution. Tribol. Internat..

[26] Zhang L.M., Ma A.L., Yu H., Umoh A.J., Zheng Y.G.S. (2019). Correlation of microstructure with cavitation erosion behaviour of a nickel-aluminum bronze in simulated seawater. Tribol. Internat..

[27] Cao L.F., Qin Z.B., Deng Y.D., Zhong C., Hu W.B., Wu Z. (2020). Effect of passive film on cavitation corrosion behavior of 316l stainless steel. Int. J. Electrochem. Sci..

[28] Qin Z.B., Cao L.F., Deng Y.D., Zhong C., Hu W.B., Wu Z. (2020). Effect of oxide film on the cavitation erosion-corrosion behavior of nickel-aluminum bronze alloy. Corrosion.

[29] Wang Y.G.Z.Z.B. (2021). Critical flow velocity phenomenon in erosion-corrosion of pipelines: Determination methods, mechanisms and applications. J. Pipeline Sci. Eng..

[30] Song Q.N., Zheng Y.G., Ni D.R., Ma Z.Y. (2015). Studies of the nobility of phases using scanning kelvin probe microscopy and its relationship to corrosion behaviour of Ni–Al bronze in chloride media. Corros. Sci..

[31] Qin Z., Zhang Q., Luo Q., Wu Z., Shen B., Liu L., Hu W. (2018). Microstructure design to improve the corrosion and cavitation corrosion resistance of a nickel-aluminum bronze. Corros. Sci..

[32] Wu Z., Cheng Y.F., Liu L., Lv W.J., Hu W.B. (2016). Effects of elastic and plastic deformations on corrosion of an aluminum bronze alloy in NaCl solution. Corrosion.

[33] Szczygiel B., Kolodziej M. (2005). Composite ni/al2o3 coatings and their corrosion resistance. Electrochim. Acta.

[34] Yu H., Zheng Y.G., Yao Z.M. (2009). Cavitation erosion corrosion behaviour of manganese-nickel-aluminum bronze in comparison with manganese-brass. J. Mater. Sci. Technol..

[35] Qian X., Gu N., Cheng Z., Yang X., Wang E., Dong S. (2001). Impedance study of (PEO)_10_LiClO_4_–Al_2_O_3_ composite polymer electrolyte with blocking electrodes. Electrochim. Acta.

[36] Xu B., Wang S.L., Li L.Q., Li S.J. (2012). Structure evolvement of solid particles and mechano-chemical effect. Acta Physica Sinica.

[37] Shuai Z., Pang X., Wang Y., Gao K. (2013). Corrosion behavior of steel with different microstructures under various elastic loading conditions. Corros. Sci..

[38] Chulist R., Straka L., Sozinov A., Tokarski T., Skrotzki W. (2017). Branched needle microstructure in ni-mn-ga 10m martensite: Ebsd study. Acta Mater..

[39] Wu R.F., Jiao Z.M., Wang Y.S., Wang Z., Wang Z.H., Ma S.G., Qiao J.W. (2016). Excellent plasticity of a new ti-based metallic glass matrix composite upon dynamic loading. Mater. Sci. Eng. A-Struct. Mater. Propert. Microstruct. Process..

[40] Imaz N., Ostra M., Vidal M., Díez J.A., Sarret M., García-Lecina E. (2014). Corrosion behaviour of chromium coatings obtained by direct and reverse pulse plating electrodeposition in nacl aqueous solution. Corros. Sci..

[41] Auret J., Damm O., Wright G., Robinson F. (1993). Influence of cathodic and anodic currents on cavitation erosion. Corrosion.

[42] Song Q.-N., Xu N., Bao Y.-F., Jiang Y.-F., Gu W., Zheng Y.-G., Qiao Y.-X. (2017). Corrosion and cavitation erosion behaviors of two marine propeller materials in clean and sulfide-polluted 3.5% nacl solutions. Acta Metall. Sin..

[43] ASTM G119-93: Standard guide for determining amount of synergism between wear, Annual book of ASTM standards, 507-512.

[44] Bai L.Y., Jiang K.B., Gao L. (2018). The influence and mechanism of residual stress on the corrosion behavior of welded structures. Mater. Res.-Ibero-Am. J. Mater..

[45] Yu H.H., Suo Z. (2000). Stress-dependent surface reactions and implications for a stress measurement technique. J. Appl. Phys..

[46] Lin J.R., Wang Z.H., Cheng J.B., Kang M., Fu X.Q., Hong S. (2017). Effect of initial surface roughness on cavitation erosion resistance of arc-sprayed fe-based amorphous/nanocrystalline coatings. Coatings.

[47] Gao H., Huang Y., Nix W.D., Hutchinson J.W. (1999). Mechanism-based strain gradient plasticity - i. Theory J. Mech. Phys. Solids.

[48] Li Y.J., Chen H.S., Chen D.R., Wang J.D. (2009). Effect of micro/nano-particles in cavitation erosion. J. Nanosci. Nanotechnol..

[49] Chen H.S., Li Y.J., Chen D.R., Wang J.D. (2007). Experimental and numerical investigations on development of cavitation erosion pits on solid surface. Tribol. Lett..

[50] Nour W.M.N., Dulias U., Schneider J., Zum Gahr K.H. (2007). The effect of surface finish and cavitating liquid on the cavitation erosion of alumina and silicon carbide ceramics. Ceram.-Silik..

[51] Decuzzi P., Demelio G.P. (2003). Stress-driven morphological instability and catastrophic failure of microdevices. Int. J. Solids Struct..

